# Gamma-glutamyl transferase to high-density lipoprotein cholesterol ratio has a non-linear association with non-alcoholic fatty liver disease: A secondary prospective cohort study in non-obese Chinese adults

**DOI:** 10.3389/fmed.2022.995749

**Published:** 2022-11-17

**Authors:** Qiming Li, Yong Han, Haofei Hu, Yuzheng Zhuge

**Affiliations:** ^1^Department of Gastroenterology, Nanjing Medical University Drum Tower Clinical Medical School, Nanjing, Jiangsu, China; ^2^Department of Emergency, Shenzhen Second People’s Hospital, Shenzhen, Guangdong, China; ^3^Department of Nephrology, Shenzhen Second People’s Hospital, Shenzhen, Guangdong, China

**Keywords:** non-alcoholic fatty liver disease, gamma-glutamyl transferase, high-density lipoprotein cholesterol, non-linear, smooth curve fitting

## Abstract

**Objective:**

The evidence for a relationship between the ratio of gamma-glutamyl transferase (GGT) to high-density lipoprotein cholesterol (HDL-c) and non-alcoholic fatty liver disease (NAFLD) is currently inadequate. This study aimed to investigate the relationship between the GGT/HDL-c ratio and NAFLD.

**Materials and methods:**

This study is a prospective cohort study that recruited a total of 11,891 non-obese volunteers in a Chinese hospital from January 2010 to December 2014 in a non-selective manner. The Cox proportional-hazards regression model was then used to investigate the relationship between baseline GGT/HDL-c ratio and the probability of developing NAFLD. The non-linear link between the GGT/HDL-c ratio and NAFLD was identified using a Cox proportional hazards regression with cubic spline functions and smooth curve fitting (cubic spline smoothing). Furthermore, we conducted several sensitivity and subgroup analyses. Data had been uploaded to the DATADRYAD website.

**Results:**

The mean age of study participants was 43.29 ± 14.95 years old, and 6,502 (54.68%) were male. The median (interquartile ranges) of GGT/HDL-c ratio was 15.56 (10.73–23.84). During a median follow-up of 29.35 months, 2028 (17.05%) participants were diagnosed with NAFLD. After adjusting for covariates, the results showed that GGT/HDL-c ratio was positively associated with incident NAFLD (HR = 1.014, 95% CI 1.011–1.017). There was also a non-linear relationship between GGT/HDL-c ratio and NAFLD, and the inflection point of the GGT/HDL-c ratio was 20.35. The effect sizes (HR) on the left and right sides of the inflection point were 1.113 (95% CI 1.096, 1.130) and 1.003 (95% CI 1.000–1.007), respectively. Moreover, the sensitivity analysis demonstrated the robustness of our results. Subgroup analysis showed that GGT/HDL-c ratio was more strongly associated with incident NAFLD in triglyceride (TG) < 1.7 mmol/L participants. In contrast, the weaker association was probed in those with TG ≥ 1.7 mmol/L.

**Conclusion:**

The present study reveals a positive and non-linear relationship between the GGT/HDL-c ratio and NAFLD risk in a non-obese Chinese population. GGT/HDL-c ratio is strongly associated with NAFLD when GGT/HDL-c ratio is less than 20.35. Therefore, maintaining the GGT/HDL-c ratio lower than the inflection point is recommended from a treatment perspective.

## Introduction

Non-alcoholic fatty liver disease (NAFLD) has surpassed alcoholic hepatitis to become the most prevalent chronic liver disease, affecting more than a quarter of all people globally (1, 2). NAFLD has gained increased recognition as the main cause of liver illness and death, imposing a significant economic burden on society (1, 3). In the United States, its prevalence is expected to increase by 33.5% by 2030 (4). Similarly, the prevalence of NAFLD in China has climbed from 15% in the early 2000s to 29.2% in 2020 (5). Fatty liver changes are a significant indicator of NAFLD, which encompasses a spectrum of liver pathologies ranging from benign simple steatosis/non-alcoholic fatty liver to hepatic inflammation and fibrosis/non-alcoholic steatohepatitis (NASH). NASH can progress to cirrhosis and hepatocellular carcinoma (6, 7). In addition, NAFLD increases the risk of developing extrahepatic metabolic abnormalities such as hypertension, hyperuricemia, dyslipidemia, hyperglycemia, and insulin resistance (IR). This eventually leads to a poor prognosis, including cardiovascular disease, type 2 diabetes mellitus (T2DM), and other metabolic compilations (8, 9). As a result, it is critical to study and monitor the prevalence and predictors of NAFLD to avoid the development of primary fatty liver disease to NASH and develop early intervention measures.

Gamma-glutamyl transferase (GGT), routinely used to assess hepatocyte damage, is an established predictor of NAFLD. It is highly related to the incidence of NAFLD (10, 11). Additionally, studies have reported that increased GGT levels are related to a more severe histological spectrum of NAFLD, such as NASH and fibrosis (12, 13). In contrast, lower high-density lipoprotein cholesterol (HDL-c) levels are a risk factor for metabolic syndrome (METS) (14, 15). NAFLD has been thought to correlate with metabolic syndrome and its components. Some regard NAFLD as a hepatic manifestation of METS (16). Additionally, in previous reports, reduced HDL-c efflux capability and anti-oxidative activity may contribute to the pathophysiology of NAFLD (17). Given the correlation between GGT and HDL-c and NAFLD, it is vital to investigate the association between the GGT/HDL-c ratio and NAFLD. However, their connection has rarely been studied. A cross-sectional study suggested that GGT/HDL-c was positively associated with the incidence of metabolic-associated fatty liver disease (18).

Moreover, past research indicates that obesity is highly connected with NAFLD (19, 20). However, it is worth mentioning that many patients with an average body mass index (BMI) nevertheless have NAFLD (21). Hepatic steatosis was present in 7.4% of non-obese adults in third National Health and Nutrition Inspection Survey of America. This percentage might range from 8 to 19% in Asia (22). Furthermore, non-obese people with NAFLD tend to be more susceptible to metabolic syndrome and have a higher chance of acquiring other significant illnesses, such as more severe liver disease, diabetes, and cardiovascular disease (23–26). Therefore, identifying non-obese individuals at risk for NAFLD may remain critical.

Unfortunately, past research has been cross-sectional with small sample size. Furthermore, none of these studies examined subgroups or the non-linear association between the GGT/HDL-c ratio and NAFLD. Additionally, the link between them in non-obese individuals is unknown. For these reasons, we examined whether the GGT/HDL-c ratio is independently linked with NAFLD in Chinese non-obese individuals with a normal range of low-density lipoprotein cholesterol (LDL-c).

## Materials and methods

### Study design

The study employed a prospective cohort design. Data were derived from a prospective, observational cohort study of a representative population created by the Wenzhou Medical Center of the Wenzhou People’s Hospital in China (27). The target-independent variable was the evaluated GGT/HDL-c ratio at baseline. The outcome variable was NAFLD (dichotomous variable: 0 = non-NAFLD, 1 = NAFLD).

### Data source

The raw data was obtained freely from the DATADRYAD database^1^ provided by Sun, Dan-Qin et al. (27). (Sun, Dan-Qin et al. data from Association of low-density lipoprotein cholesterol within the normal range and NAFLD in the non-obese Chinese population: a cross-sectional and longitudinal study, Dryad, Dataset, https://doi.org/10.5061/dryad.1n6c4). Under Dryad’s terms of service, researchers could use this data for secondary analyses without violating authors’ rights.

### Study population

The original researchers initially recruited NAFLD-free subjects who underwent a health examination at Wenzhou Medical Center of Wenzhou People’s Hospital in a non-selective and sequential manner (27). The original study was conducted with approval from the ethics committee of Wenzhou People’s Hospital (27). All participants have given informed consent to participate in the study. Therefore, the current secondary analysis has not received ethical approval (27). Additionally, the original study was done in compliance with the Helsinki Declaration. All processes were carried out following applicable standards and regulations, as indicated in the Declarations section. So did this secondary analysis.

**FIGURE 1:**
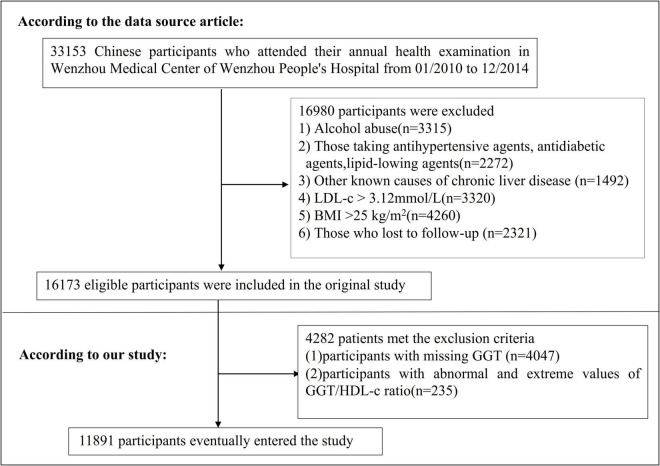
Flowchart of study participants.

The study initially considered 33,153 NAFLD-free non-obese individuals; 16,980 participants were excluded. A total of 16,173 participants who completed the 5-year follow-up were considered for eligibility in the study (Figure 1; 27). The original study’s inclusion criteria were NAFLD-free Chinese adults in longitudinal studies who have had a health assessment between January 2010 and December 2014. There were several exclusion criteria: (i) those with excessive alcohol consumption (per week ≥ 140 g for males and ≥ 70 g/week for females); (ii) those with any known causes of chronic hepatic diseases, such as NAFLD, autoimmune hepatitis, or viral hepatitis; (iii) those with BMI ≥ 25 kg/m^2^ and LDL-c > 3.12 mmol/L; (iv) those taking antihypertensive, lipid-lowering, or antidiabetic agents; (v) those who were lost to follow-up or their data were missing (27). In the current study, we further excluded participants with missing GGT values (*n* = 4,047) and participants with abnormal and extreme values of GGT/HDL-c ratio (more significant or less than three standard deviations from the mean) were also excluded (*n* = 235) (28). Finally, our study comprised 11,891 individuals.

### Variables

The GGT/HDL-c ratio was recorded as a continuous variable. The detailed process of defining the GGT/HDL-c ratio was described: GGT/HDL-c ratio = serum GGT divided by HDL-c. It should be noted that the unit of GGT and HDL-c was U/L and mmol/L, respectively.

### Outcome measures

Our outcome variable was NAFLD (dichotomous variable: 0 = non-NAFLD, 1 = NAFLD). As per recommendation by the Chinese Liver Disease Association, NAFLD was diagnosed by ultrasonography based on the following criteria: (a) diffusely enhanced near-field echoes in the liver area and gradually attenuated far-field echoes; (b) unclear intrahepatic cavity structure; (c) mild to moderate hepatomegaly, with rounded edges; (d) decreased blood flow signal in the liver; (e) poorly or incompletely visualized right hepatic lobe and diaphragmatic capsule (27).

Annual follow-up assessments were performed during the observation period. Liver ultrasonography was performed in a blinded (as at baseline) manner to determine the incidence of NAFLD. Participants were censored at the time of diagnosis of NAFLD or the last visit, whichever came first. The follow-up period was 5 years.

### Covariates

Covariates were selected in our study according to our clinical experience and the previous literature. As a result of the preceding, the continuous and categorical variables were regarded as covariates. Continuous variables included age, BMI, systolic blood pressure (SBP), alanine aminotransferase (ALT), diastolic blood pressure (DBP), albumin (ALB), aspartate aminotransferase (AST), globulin (GLB), γ-glutamyl transpeptidase (GGT), direct bilirubin (DBIL), LDL-c, alkaline phosphatase (ALP), uric acid (UA), total bilirubin (TBIL), serum triglyceride (TG), blood urea nitrogen (BUN), HDL-c, fasting plasma glucose (FPG), and total serum cholesterol (TC), and estimated glomerular filtration rate (eGFR). Categorical variables included gender.

All biochemical parameters were assayed *via* automated analyzer (Abbott AxSYM). A physician took a health habit inventory and medical history (27). BMI was computed by dividing the weight in kilos by the height in meters squared (kg/m^2^). Data was collected under standardized conditions and processed according to a uniform process (27). According to the World Health Organization (WHO), impaired fasting glucose (IFG) was defined as FPG of 6.1–6.9 mmol/L (29). FPG ≥ 7 mmol/L was defined as diabetes (30). ALT > 40U/L reflected liver dysfunction (31). Hypertriglyceridemia (HTG) refers to serum TG levels ≥ 1.7 mmol/L (32). The eGFR was determined using the Chronic Kidney Disease Epidemiology Collaboration’s (CKD-EPI) algorithm for “Asian origin” (33). Previous reports provide more detailed information (27).

### Missing data processing

In observational research, missing data is a regular occurrence that can never be completely prevented (34). In our study, the numbers of participants with missing data for BUN, Scr, UA, FBG, SBP, DBP, ALB, GLB, TBIL, DBIL were 1 (0.01%), 1 (0.01%), 1 (0.01%), 1 (0.01%), 16 (0.13%), 16 (0.13%), 1379 (11.60%), 1379 (11.60%), 3170 (26.66%), and 4581 (38.52%), respectively. To mitigate the variation caused by missing variables, which cannot accurately reflect the statistical efficiency of the target sample throughout the modeling phase, this study used multiple imputations for missing data (34, 35). The imputation model included Sex, Age, SBP, DBP, AST, GLB, ALP, BMI, ALB, DBIL, BUN, TG, ALT, FBG, TB, UA, and LDL-c.

### Statistical analysis

We stratified the participants by quartiles of GGT/HDL-c ratio. Mean ± standard deviation (SD) (Gaussian distribution) or median (interquartile ranges) (skewed distribution) were reported for continuous variables, and frequencies and percentages were presented for categorical variables. We used χ2 (categorical variables), the one-way ANOVA test (normal distribution), or the Kruskal-Wallis *H* test (skewed distribution) to test for differences among different GGT/HDL-c ratios groups.

To explore the connection between the GGT/HDL-c ratio and NAFLD, we used univariate and multivariate Cox proportional-hazards regression models, including a non-adjusted model (Crude model; no covariates were adjusted), minimally adjusted model (Model I, adjusted age, gender, SBP, BMI, and DBP) and a fully adjusted model (Model II, adjusted age, sex, SBP, BMI, DBP, ALT, ALP, ALB, TBIL, GLB, UA, FBG, TG, Scr, LDL-c). Effect sizes (HR) with 95% confidence intervals (CI) were recorded. We adjusted them when the covariances were added to the model, and the hazard ratio (HR) changed by 10% or greater (36). Additionally, it alluded to the collinearity screening findings. Collinearity screening revealed that TC and DBIL were collinear with other variables and were not included in the final multivariate logistic regression equation.

We also employed a Cox proportional hazards regression model with cubic spline functions and smooth curve fitting to account for the non-linear connection between GGT/HDL-c and NAFLD. Moreover, the two-piecewise Cox proportional-hazards regression model was employed to elucidate the non-linear relationship between the GGT/HDL-c ratio and NAFLD (37). Finally, a log-likelihood ratio test determined the best acceptable model for characterizing the risk associated with the GGT/HDL-c ratio and NAFLD.

Subgroup analyses across multiple subgroups were conducted using a stratified Cox proportional-hazards regression model (gender, FPG, age, BMI, TG, ALT, SBP, DBP, and UA). Firstly, continuous variable age (< 30, ≥ 30 to < 40, ≥ 40 to < 50, ≥ 50 to < 60, ≥ 60 to < 70, ≥ 70 years), BMI (< 18.5, ≥ 18.5 to < 24, ≥ 24 kg/m^2^), FPG (≤ 6.1, > 6.1 mmol/L), ALT (≤ 40, > 40 U/L), SBP (< 140, ≥ 140 mmHg), DBP (< 90, ≥ 90 mmHg), TG (< 1.7, ≥ 1.7 mmol/L), UA (< 420, ≥ 420 g/L) (38) were converted to a categorical variable based on the clinical cut point. Secondly, in addition to the stratification factor itself, we adjusted each stratification for all factors (age, sex, SBP, BMI, DBP, ALT, ALP, GLB, TBIL, ALB, UA, FBG, TG, Scr, LDL-c). Finally, the likelihood ratio test was used to determine the presence or absence of interaction terms in models with and without interaction terms (39).

To test the robustness of our results, we performed a series of sensitivity analyses. We converted the GGT/HDL-c ratio into a categorical variable according to the quartile and calculated the P for the trend to test the results of the GGT/HDL-c ratio as the continuous variable and to explore the possibility of non-linearity. Diabetes (40), TG (41), ALT (42), and chronic kidney disease (43) are significantly associated with NAFLD. Therefore, when exploring the association between TG/HDL-c and incident NAFLD in other sensitivity analyses, we excluded participants with FPG > 6.1 mmol/L, TG ≥ 1.7 mmol/L, ALT > 40 U/L, or eGFR < 60 mL/min^⋅^1.73 m^2^. Besides, we also used a generalized additive model (GAM) to insert the continuity covariate into the equation (model III) as a curve to ensure the robustness of the results (44). In addition, we explored the potential for unmeasured confounding between GGT/HDL-c ratio and NAFLD risk by calculating *E*-values (45). All results were written in accordance with the STROBE guidelines (46).

All analyses were conducted using R’s statistical software packages (The R Foundation; Vienna, Austria) and EmpowerStats (X&Y Solutions, Inc., Boston, MA, USA). Statistical significance was defined as *P* values less than 0.05 (two-sided).

## Results

### Characteristics of participants

Table 1 provides the demographic and clinical characteristics of participants included in the study. The mean age was 43.29 ± 14.95 years, and 6502 (54.68%) were male. The median (interquartile ranges) of GGT/HDL-c ratio was 15.56 (10.73–23.84). During 29.35 months, 2028 (17.05%) people experienced NAFLD during a median follow-up time. We assigned the adults into subgroups using GGT/HDL-c ratio quartiles (< 10.72, 10.72–15.55, 15.55–23.85, ≥ 23.85). When compared with the Q1 group (< 10.72), age, ALP, GGT, GGT/HDL-c ratio, ALT, AST, GLB, TBIL, Scr, BUN, UA, TC, FPG, TG, BMI, SBP, DBP increased significantly in the Q4 group (≥ 23.85), while the opposite results were found in covariates in terms of male, HDL-c.

**TABLE 1:** The baseline characteristics of participants.

GGT/HDL-c ratio (quartile)	Q1 (< 10.72)	Q2 (10.72–15.55)	Q3 (15.55–23.85)	Q4 (≥ 23.85)	*P*-value
participants	2,971	2,971	2,976	2,973	
Gender					< 0.001
Female	1503 (50.59%)	1359 (45.74%)	1302 (43.75%)	1225 (41.20%)	
Male	1468 (49.41%)	1612 (54.26%)	1674 (56.25%)	1748 (58.80%)	
Age (years)	42.52 ± 14.92	43.07 ± 15.00	43.34 ± 15.05	44.24 ± 14.80	< 0.001
ALP (U/L)	63.18 ± 18.26	69.57 ± 19.71	74.41 ± 20.83	80.15 ± 25.73	< 0.001
GGT (U/L)	14.65 ± 3.07	19.31 ± 3.97	25.23 ± 5.94	48.89 ± 23.37	< 0.001
HDL-c (mmol/L)	1.75 ± 0.31	1.49 ± 0.29	1.32 ± 0.28	1.24 ± 0.31	< 0.001
GGT/HDL-c ratio	8.67 (7.43–9.72)	12.93 (11.80–14.20)	18.89 (17.04–20.96)	34.02 (27.87–46.20)	< 0.001
ALT (U/L)	13.00 (10.00–16.00)	15.00 (11.00–19.00)	17.00 (14.00–23.00)	23.00 (17.00–31.00)	< 0.001
AST (U/L)	19.97 ± 4.95	21.54 ± 6.82	22.83 ± 6.81	26.57 ± 13.07	< 0.001
ALB (g/L)	44.29 ± 2.75	44.54 ± 2.74	44.70 ± 2.81	44.64 ± 2.78	< 0.001
GLB (g/L)	28.98 ± 3.96	29.26 ± 3.92	29.37 ± 4.02	29.53 ± 4.02	< 0.001
TBIL (umol/L)	11.59 ± 4.69	12.16 ± 4.91	12.74 ± 5.07	12.57 ± 5.43	< 0.001
DBIL (umol/L)	2.00 (1.40–2.64)	2.10 (1.46–2.86)	2.10 (1.50–2.90)	2.00 (1.40–2.80)	< 0.001
BUN (umol/L)	4.32 ± 1.33	4.57 ± 1.40	4.69 ± 1.30	4.76 ± 1.57	< 0.001
Scr (umol/L)	74.00 ± 16.44	81.87 ± 21.99	87.24 ± 19.96	90.60 ± 36.38	< 0.001
UA (umol/L)	234.79 ± 74.91	277.92 ± 81.28	312.67 ± 79.16	341.25 ± 81.14	< 0.001
FPG (mmol/L)	5.04 ± 0.58	5.14 ± 0.72	5.23 ± 0.81	5.40 ± 1.08	< 0.001
TC (mmol/L)	4.62 ± 0.72	4.53 ± 0.73	4.56 ± 0.72	4.69 ± 0.75	< 0.001
TG (mmol/L)	0.85 (0.69–1.06)	1.02 (0.81–1.31)	1.28 (0.99–1.66)	1.64 (1.20–2.31)	< 0.001
LDL-c (mmol/L)	2.17 ± 0.47	2.24 ± 0.47	2.33 ± 0.46	2.35 ± 0.46	< 0.001
BMI (kg/m2)	20.59 ± 1.94	21.24 ± 2.00	21.95 ± 1.93	22.58 ± 1.72	< 0.001
SBP (mmHg)	115.60 ± 15.92	120.73 ± 16.50	124.75 ± 16.34	127.78 ± 16.40	< 0.001
DBP (mmHg)	69.71 ± 9.40	72.76 ± 10.03	74.93 ± 9.97	77.25 ± 10.41	< 0.001

Values are n (%) or mean ± SD or median (quartile).

BMI, Body mass index; DBP, Diastolic blood pressure; ALP, Alkaline phosphatase; SBP, Systolic blood pressure; GGT, γ-glutamyl transpeptidase; AST, Aspartate aminotransferase; TG, Triglyceride; ALB, albumin; ALT, Alanine aminotransferase; GLB, globulin; LDL-c, Low-density lipid cholesterol; BUN, Serum urea nitrogen; HDL-c, High-density lipoprotein cholesterol; Scr, Serum creatinine; TC, Total cholesterol; FPG, fasting plasma glucose; UA, uric acid; eGFR, evaluated glomerular filtration rate; DBIL, Direct bilirubin; TBIL, total bilirubin; GGT/HDL-c ratio, Gamma-glutamyl transpeptidase to high-density lipoprotein cholesterol ratio.

**FIGURE 2:**
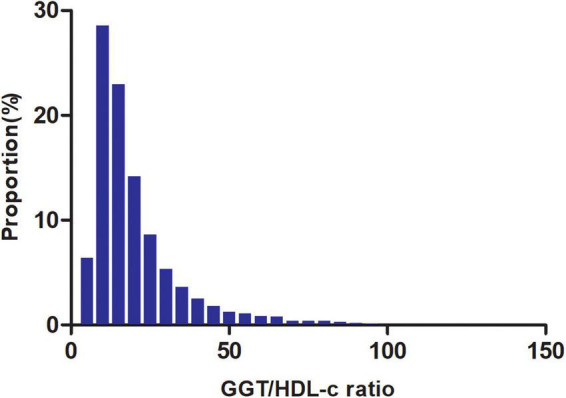
Distribution of GGT/HDL-c ratio. It presented a skewed distribution while being in the range from 2.72 to 95.95, with a median of 15.56.

**FIGURE 3:**
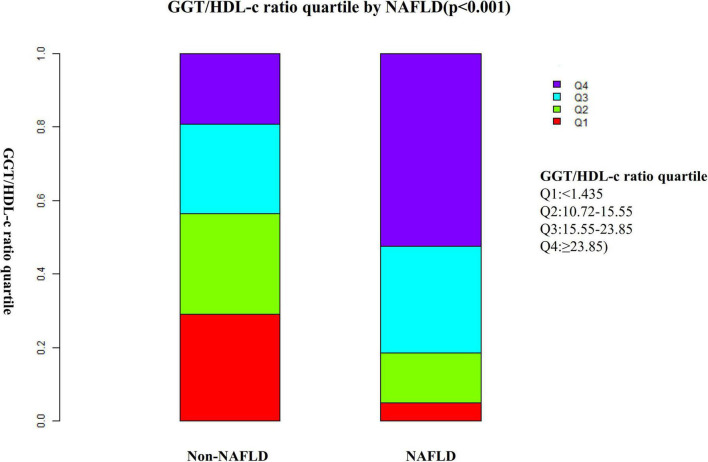
Data visualization of GGT/HDL-c ratio of all participants from the non-alcoholic fatty liver disease (NAFLD) and non-NAFLD groups. Indicated that the non-alcoholic fatty liver disease (NAFLD) group’s distribution GGT/HDL-c ratio was higher. In contrast, the GGT/HDL-c ratio in the non-NAFLD group was relatively lower.

**FIGURE 4:**
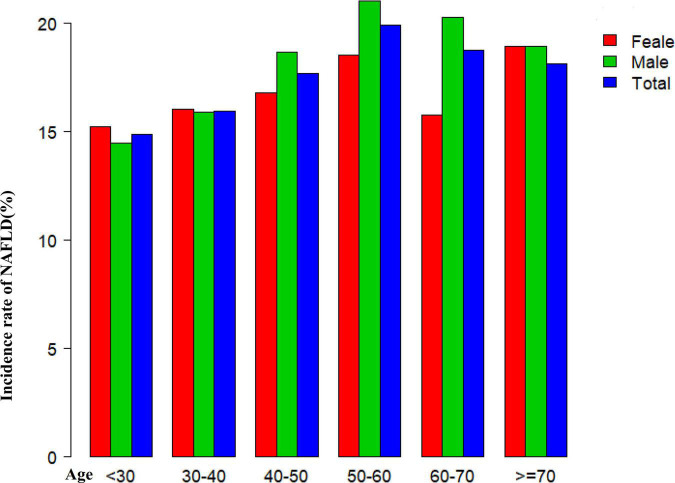
Non-alcoholic fatty liver disease (NAFLD) incidence rate of age stratification by ten intervals. Showed that in age stratification by ten intervals, among participants aged 40–70, the incidence of NAFLD was higher in males than in females, regardless of their age group (Figure 4). It also found that the incidence of NAFLD increased with age, both in males (except for age > 60 years) and females (except 60–70 years old) participants.

Figure 2 shows the distribution of the GGT/HDL-c ratio. It presented a skewed distribution with the range from 2.72 to 95.95, and a median of 15.56. Participants were divided into two groups according to whether they experienced NAFLD. The GGT/HDL-c ratio in the two groups is shown in Figure 3. The results indicated that the distribution level of the GGT/HDL-c ratio in the non-NAFLD group was lower. In contrast, the GGT/HDL-c ratio in the NAFLD group was relatively higher. In participants aged 40–70 years, the incidence of NAFLD was higher in males than in females, regardless of their age group (Figure 4). We also observed that the incidence of NAFLD increased with age, both in males (except for age > 60 years) and females (except 60–70 years old).

### The incidence rate of non-alcoholic fatty liver disease

Table 2 revealed that 2028 (17.05%) participants developed NAFLD during a median follow-up time of 29.35 months. The total cumulative incidence rate of all persons was 6.99 per 100 person-years. In particular, the cumulative incidence of the four GGT/HDL-c ratio groups was 1.41, 3.79, 7.94, and 14.65 per 100 person-years, respectively. The incidence rate of total NAFLD and each GGT/HDL-c ratio group was 17.05% (16.39–17.73%), 3.30% (2.66–3.94%), 9.32% (8.28–10.37%), 19.69% (18.26–21.12%), and 35.89% (34.16–37.61%), respectively. Participants with a high GGT/HDL-c ratio had higher NAFLD incidence rates than the group with the lowest GGT/HDL-c ratio (*p* < 0.001 for trend) (Supplementary Figure 1).

**TABLE 2:** Incidence rate of non-alcoholic fatty liver disease (NAFLD) (%).

GGT/HDL-c ratio	Participants (n)	NAFLD events (n)	Incidence rate (95% CI) (%)	Per 100 person-year
Total	11891	2028	17.05 (16.39–17.73)	6.99
Q1 (< 10.72)	2971	98	3.30 (2.66–3.94)	1.41
Q2 (10.72–15.55)	2971	277	9.32 (8.28–10.37)	3.79
Q3 (15.55–23.85)	2976	586	19.69 (18.26–21.12)	7.94
Q4 (≥ 23.85)	2973	1067	35.89 (34.16–37.61)	14.65
P for trend			< 0.001	< 0.001

GGT/HDL-c ratio, Gamma-glutamyl transpeptidase to high-density lipoprotein cholesterol ratio; NAFLD, non-alcoholic fatty liver disease; CI, confidence interval.

### The results of univariate analyses using Cox proportional-hazards regression model

The univariate analyses showed that non-alcoholic fatty liver disease had nothing to do with ALB, gender (all *P* > 0.05), but was positively related to age (HR = 1.006, 95% CI 1.003, 1.009), ALP (HR = 1.009, 95% CI 1.008, 1.010), GGT (HR = 1.017, 95% CI 1.016, 1.019), GGT/HDL-c ratio (HR = 1.030, 95% CI 1.028, 1.032), ALT (HR = 1.007, 95% CI 1.006, 1.008), AST (HR = 1.008, 95% CI 1.006, 1.011), GLB (HR = 1.019, 95% CI 1.008, 1.030), Scr (HR = 1.003, 95% CI 1.001, 1.004), UA (HR = 1.002, 95% CI 1.002, 1.003), FPG (HR = 1.219, 95% CI 1.186, 1.253), TC (HR = 1.307, 95% CI 1.232, 1.386), TG (HR = 1.414, 95% CI 1.384, 1.445), LDL-c (HR = 1.799, 95% CI 1.628, 1.988), BMI (HR = 1.679, 95% CI 1.629, 1.730), SBP (HR = 1.014, 95% CI 1.012, 1.016), DBP (HR = 1.033, 95% CI 1.029, 1.037), and negatively related to HDL-c (HR = 0.285, 95% CI 0.249, 0.327), TBIL (HR = 0.984, 95% CI 0.976, 0.993), DBIL (HR = 0.730, 95% CI 0.702, 0.758), BUN (HR = 0.876, 95% CI 0.847, 0.907) (all *P* < 0.05; Table 3).

**TABLE 3:** The results of the univariate Cox proportional hazards model.

Exposure	Statistics	HR (95% CI)	*P*-value
**Gender**			
Male	6502 (54.680%)	Ref	
Female	5389 (45.320%)	1.055 (0.966, 1.152)	0.23380
Age (years)	43.293 ± 14.953	1.006 (1.003, 1.009)	0.00006
ALP (U/L)	71.831 ± 22.208	1.009 (1.008, 1.010)	< 0.00001
GGT (U/L)	27.023 ± 18.032	1.017 (1.016, 1.019)	< 0.00001
HDL-c (mmol/L)	1.451 ± 0.357	0.285 (0.249, 0.327)	< 0.00001
GGT/HDL-c ratio	20.044 ± 14.312	1.030 (1.028, 1.032)	< 0.00001
ALT (U/L)	19.550 ± 15.365	1.007 (1.006, 1.008)	< 0.00001
AST (U/L)	22.730 ± 8.830	1.008 (1.006, 1.011)	< 0.00001
ALB (g/L)	44.543 ± 2.773	0.994 (0.979, 1.010)	0.46073
GLB (g/L)	29.284 ± 3.987	1.019 (1.008, 1.030)	0.00060
TBIL (umol/L)	12.264 ± 5.053	0.984 (0.976, 0.993)	0.00051
DBIL (umol/L)	2.206 ± 1.196	0.730 (0.702, 0.758)	< 0.00001
BUN (umol/L)	4.585 ± 1.413	0.876 (0.847, 0.907)	< 0.00001
Scr (umol/L)	83.431 ± 25.654	1.003 (1.001, 1.004)	< 0.00001
UA (umol/L)	291.675 ± 88.578	1.002 (1.002, 1.003)	< 0.00001
FPG (mmol/L)	5.205 ± 0.827	1.219 (1.186, 1.253)	< 0.00001
TC (mmol/L)	4.601 ± 0.731	1.307 (1.232, 1.386)	< 0.00001
TG (mmol/L)	1.346 ± 0.835	1.414 (1.384, 1.445)	< 0.00001
LDL-c (mmol/L)	2.272 ± 0.470	1.799 (1.628, 1.988)	< 0.00001
BMI (kg/m2)	21.590 ± 2.039	1.679 (1.629, 1.730)	< 0.00001
SBP (mmHg)	122.216 ± 16.916	1.014 (1.012, 1.016)	< 0.00001
DBP (mmHg)	73.664 ± 10.337	1.033 (1.029, 1.037)	< 0.00001

Values are n (%) or mean ± SD.

BMI, Body mass index; SBP, Systolic blood pressure; ALP, Alkaline phosphatase; DBP, Diastolic blood pressure; GGT, γ-glutamyl transpeptidase; TC, Total cholesterol; ALT, Alanine aminotransferase; Scr, Serum creatinine; LDL-C, Low-density lipid cholesterol; AST, Aspartate aminotransferase; ALB, albumin; GLB, globulin; TG, Triglyceride; UA, uric acid; HDL-C, High-density lipoprotein cholesterol; BUN, Serum urea nitrogen; FPG, Fasting plasma glucose; DBIL, Direct bilirubin; TBIL, Total bilirubin; GGT/HDL-c ratio, Gamma -glutamyl transpeptidase to high-density lipoprotein cholesterol ratio; HR, Hazard ratios; CI, confidence interval; Ref, reference.

**FIGURE 5:**
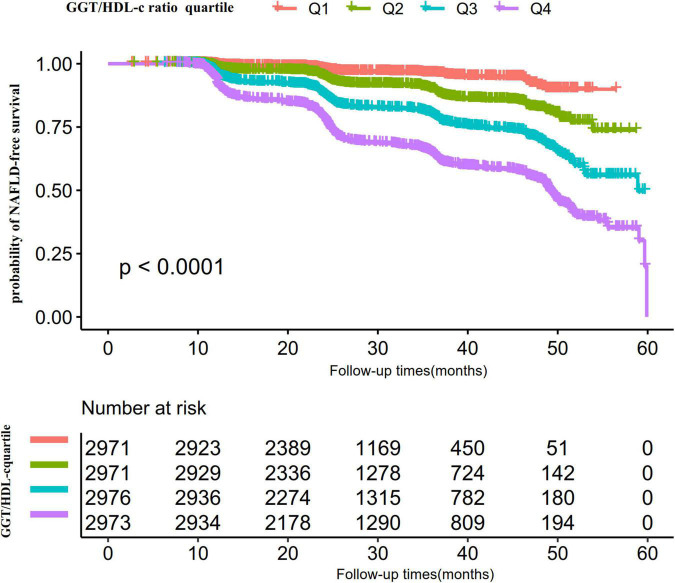
Kaplan–Meier event-free survival curve. The probability of non-alcoholic fatty liver disease (NAFLD)-free survival differed significantly between the GGT/HDL-c ratio quartiles (log-rank test, *p* < 0.001). The probability of NAFLD-free survival gradually increased with increasing GGT/HDL-c ratio, suggesting that the group with the highest GGT/HDL-c ratio had the highest risk of NAFLD.

Kaplan-Meier survival curves for NAFLD-free survival probability stratified by the GGT/HDL-c ratio group are shown in Figure 5. There were significant differences in the probability of NAFLD-free survival between the GGT/HDL-c groups (log-rank test, *P* < 0.0001). The probability of NAFLD-free survival gradually decreased with increasing GGT/HDL-c ratio, indicating that the group with the highest GGT/HDL-c ratio had the highest risk of NAFLD.

### The results of multivariate analyses using Cox proportional-hazards regression model

The authors constructed three models using the Cox proportional-hazards regression model to investigate the relationship between GGT/HDL-c ratio and incident NAFLD (Table 4). In the unadjusted model (Crude model), an increase of 1 unit of GGT/HDL-c ratio was linked with a 3% increase in the risk of NAFLD (HR = 1.30, 95% CI = 1.028, 1.032, *P* < 0.001). In the minimally-adjusted model (Model I), when we only adjusted for demographic variables, each additional 1 unit of GGT/HDL-c ratio increased by 2.1% in the risk of NAFLD (HR = 1.021, 95% CI 1.018–1.023, *p* < 0.001). In the fully-adjusted model (Model II), each additional 1 unit of GGT/HDL-c ratio was accompanied by a 1.4% increase in the risk of NAFLD (HR = 1.014, 95% CI 1.011–1.017). The distribution of confidence intervals indicates that the relationship between GGT/HDL-c ratio and NAFLD obtained by the model was reliable.

**TABLE 4:** Relationship between GGT/HDL-c ratio and the incident non-alcoholic fatty liver disease (NAFLD) in different models.

Exposure	Crude model I (HR, 95% CI, *P*)	Model I (HR, 95% CI, *P*)	Model II (HR, 95% CI, *P*)	Model III (HR, 95% CI, *P*)
GGT/HDL-c ratio	1.030 (1.028, 1.032) < 0.001	1.021 (1.018, 1.023) < 0.001	1.014 (1.011, 1.017) < 0.001	1.007 (1.004, 1.010) < 0.001
**GGT/HDL-c ratio (quartile)**				
Q1	Ref	Ref	Ref	Ref
Q2	2.531 (2.010, 3.187) < 0.001	1.943 (1.542, 2.449) < 0.001	1.924 (1.525, 2.428) < 0.001	1.551 (1.225, 1.965) < 0.001
Q3	5.203 (4.200, 6.445) < 0.001	3.244 (2.613, 4.027) < 0.001	3.006 (2.412, 3.746) < 0.001	2.001 (1.592, 2.514) < 0.001
Q4	9.542 (7.755, 11.739) < 0.001	4.864 (3.938, 6.008) < 0.001	3.925 (3.145, 4.900) < 0.001	2.311 (1.830, 2.919) < 0.001
P for trend	< 0.001	< 0.001	< 0.001	< 0.001

Crude model: we did not adjust other covariates.

Model I: we adjusted age, DBP, sex, BMI, SBP.

Model II: we adjusted age, sex, SBP, BMI, DBP, ALT, ALP, ALB, TBIL, GLB, UA, FBG, TG, Scr, and LDL-c.

Model III: we adjusted age (smooth), sex, SBP (smooth), BMI (smooth), DBP (smooth), ALT (smooth), ALP (smooth), ALB (smooth), TBIL (smooth), TG (smooth), UA (smooth), FBG (smooth), TG (smooth), Scr, LDL-c (smooth).

HR, Hazard ratios; CI, confidence; Ref, reference; NAFLD, non-alcoholic fatty liver disease.

### Sensitivity analysis

A series of sensitivity analyses were performed to verify our findings’ robustness. We first transformed the GGT/HDL-c ratio from a continuous variable to a categorical variable (according to quartiles) and then put the categorically changed GGT/HDL-c ratio back into the regression equation. The results showed that the trends in effect sizes (HR) between groups were equidistant after transforming GGT/HDL-c ratio into a categorical variable. P for the trend was consistent with the result when GGT/HDL-c ratio was a continuous variable.

Additionally, we utilized a GAM to introduce the continuity covariate as a curve into the equation. Model III’s outcome in Table 4 demonstrated that this remained reasonably consistent with the fully corrected model (HR = 1.007, 95% CI: 1.004, 1.010, < 0.001). Besides, we generated an *E*-value to assess the sensitivity to unmeasured confounding. The *E*-value (1.11) was more significant than the relative risk (1.06) of unmeasured confounders and GGT/HDL-c ratio, suggesting unmeasured or unknown confounders had little effect on the relationship between GGT/HDL-c ratio and incident NAFLD.

Furthermore, we excluded participants with FPG > 6.1 mmol/L in other sensitivity analyses. After correcting for confounding variables, the findings indicated that GGT/HDL-c ratio was also positively associated with NAFLD risk (HR = 1.014, 95% CI: 1.011–1.017, *P* < 0.001) (Table 5). For sensitivity analyses, we also excluded participants with ALT > 40 U/L (HR = 1.009, 95% CI: 1.006–1.012, *P* < 0.001), TG ≥ 1.7 mmol/L (HR = 1.013, 95% CI: 1.009–1.018, *P* < 0.001) or eGFR < 60 mL/min^⋅^1.73 m^2^ (HR = 1.014, 95% CI: 1.011–1.017, *P* < 0.001). We obtained similar results. The results of all sensitivity studies demonstrated the robustness of our findings (Table 5).

**TABLE 5:** Relationship between GGT/HDL-c ratio and non-alcoholic fatty liver disease (NAFLD) in different sensitivity analyses.

Exposure	Model I (HR, 95% CI, *P*)	Model II (HR, 95% CI, *P*)	Model III (HR, 95% CI, *P*)	Model IV (HR, 95% CI, *P*)
GGT/HDL-c ratio	1.014 (1.011, 1.017) < 0.001	1.009 (1.006, 1.012) < 0.001	1.013 (1.009, 1.018) < 0.001	1.014 (1.011, 1.017) < 0.001
**GGT/HDL-c ratio (quartile)**				
Q1	Ref	Ref	Ref	Ref
Q2	1.870 (1.466, 2.385) < 0.001	1.809 (1.432, 2.286) < 0.001	1.857 (1.449, 2.380) < 0.001	1.931 (1.521, 2.452) < 0.001
Q3	2.943 (2.338, 3.705) < 0.001	2.635 (2.107, 3.294) < 0.001	2.604 (2.045, 3.317) < 0.001	2.933 (2.339, 3.678) < 0.001
Q4	3.972 (3.151, 5.008) < 0.001	3.134 (2.491, 3.941) < 0.001	3.147 (2.449, 4.044) < 0.001	3.845 (3.060, 4.832) < 0.001
P for trend	< 0.001	< 0.001	< 0.001	< 0.001

Model I was sensitivity analysis in participants without FPG > 6.1 mmol/L (*N* = 11066). We adjusted age, sex, SBP, BMI, DBP, ALT, ALP, ALB, TBIL, GLB, UA, FBG, TG, Scr, and LDL-c.

Model II was sensitivity analysis in participants without ALT > 40 U/L (*N* = 11308). We adjusted age, sex, SBP, BMI, DBP, ALT, ALP, ALB, TBIL, GLB, UA, FBG, TG, Scr, and LDL-c.

Model III was sensitivity analysis in participants without TG ≥ 1.7 mmol/L (*N* = 9446). We adjusted age, sex, SBP, BMI, DBP, ALT, ALP, ALB, TBIL, GLB, UA, FBG, TG, Scr, and LDL-c.

Model IV was sensitivity analysis in participants without eGFR < 60 mL/min^⋅^1.73 m^2^ (*N* = 10744). We adjusted age, sex, SBP, BMI, DBP, ALT, ALP, ALB, TBIL, GLB, UA, FBG, TG, Scr, and LDL-c.

HR, Hazard ratios; CI, confidence; Ref, reference; eGFR, evaluated glomerular filtration rate (mL/min^⋅^1.73 m^2^); NAFLD, non-alcoholic fatty liver disease.

### Cox proportional hazards regression model with cubic spline functions to address non-linearity

**FIGURE 6:**
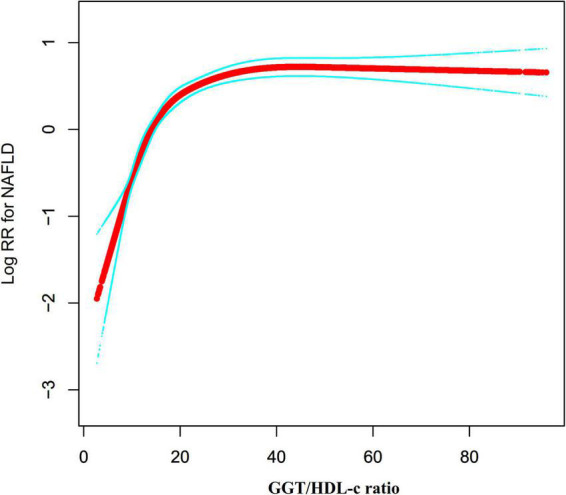
The non-linear relationship between the GGT/HDL-c ratio and the risk of non-alcoholic fatty liver disease (NAFLD). We used a Cox proportional hazards regression model with cubic spline functions to evaluate the relationship between GGT/HDL-c ratio and NAFLD risk. The result showed that the relationship between GGT/HDL-c ratio and NAFLD was non-linear, with the inflection point of the GGT/HDL-c ratio being 20.35.

We noticed that the link between GGT/HDL-c ratio and NAFLD was non-linear using the Cox proportional hazards regression model with cubic spline functions (Figure 6). As a result, data were fitted to a piecewise binary logistic regression model to account for the two distinct slopes. Additionally, we fitted data using a typical binary logistic regression model and selected the best-suited model using the log-likelihood ratio test (Table 6). The P for the log-likelihood ratio test was < 0.001. We first determined the inflection point of the GGT/HDL-c ratio (20.35) using a recursive technique and then estimated the HR and CI on both sides of the inflection point using a two-piecewise Cox proportional-hazards regression model. The HR was 1.113 on the left side of the inflection point (95% CI: 1.096, 1.130). The HR was 1.003 on the right side of the inflection point (95% CI: 1.000–1.007).

**TABLE 6:** The result of the two-piecewise Cox regression model.

Incident NAFLD	HR, 95% CI *P*
Fitting model by standard Cox regression	1.014 (1.011, 1.017) < 0.001
Fitting model by two-piecewise Cox regression	
Inflection point of GGT/HDL-c ratio	20.35
**≤ 20.35**	1.113 (1.096, 1.130) < 0.001
**> 20.35**	1.003 (1.000, 1.007) 0.0401
P for log-likelihood ratio test	< 0.001

HR, Hazard ratios; CI, confidence; Ref, reference. we adjusted age, sex, SBP, BMI, DBP, ALT, ALP, ALB, TBIL, GLB, UA, FBG, TG, Scr, and LDL-c.

### The results of subgroup analyses

In all of the prespecified or exploratory subgroups evaluated (Table 7), there was no significant interaction in age, gender, FPG, BMI, UA, ALT, DBP, and SBP. In contrast, significant interactions were detected in TG. More precisely, a greater correlation between GGT/HDL-c ratio and NAFLD was observed in TG < 1.7 mmol/L participants (HR = 1.018, 95% CI: 1.014–1.021, *p* < 0.001). In contrast, the weaker association was probed in those with TG ≥ 1.7 mmol/L (HR = 1.012, 95% CI: 1.008–1.015, *P* < 0.001).

**TABLE 7:** Effect size of GGT/HDL-c ratio on non-alcoholic fatty liver disease (NAFLD) in prespecified and exploratory subgroups.

Characteristic	No. of participants	HR (95% CI)	*P*-value	P for interaction
Age, years				0.7997
< 30	2245	1.010 (1.003, 1.018)	0.0071	
30 to < 40	3531	1.014 (1.009, 1.019)	< 0.0001	
40 to < 50	2699	1.014 (1.008, 1.020)	< 0.0001	
50 to < 60	1576	1.014 (1.007, 1.021)	0.0001	
60 to < 70	826	1.007 (0.997, 1.017)	0.1661	
≥ 70	1014	1.013 (1.005, 1.022)	0.0027	
Gender				0.0869
Male	6502	1.016 (1.012, 1.019)	< 0.0001	
Female	5389	1.012 (1.008, 1.015)	< 0.0001	
BMI (kg/m^2^)				0.2277
< 18.5	946	1.001 (0.890, 1.126)	0.9873	
≥ 18.5, < 24	9368	1.018 (1.015, 1.022)	< 0.0001	
≥ 24	1577	1.013 (1.008, 1.018)	< 0.0001	
FPG (mmol/L)				0.0874
≤ 6.1	11066	1.015 (1.012, 1.018)	< 0.0001	
> 6.1	825	1.008 (1.000, 1.016)	0.0409	
TG (mmol/L)				0.0184
< 1.7	9404	1.018 (1.014, 1.021)	< 0.0001	
≥ 1.7	2487	1.012 (1.008, 1.015)	< 0.0001	
ALT (U/L)				0.1284
≤ 40	11308	1.015 (1.012, 1.018)	< 0.0001	
> 40	583	1.010 (1.003, 1.016)	0.0034	
UA (umol/L)				0.5161
< 420	10958	1.014 (1.011, 1.017)	< 0.0001	
≥ 420	933	1.011 (1.004, 1.018)	0.0013	
SBP (mmHg)				0.0521
< 140	10177	1.015 (1.012, 1.018)	< 0.0001	
≥ 140	1714	1.010 (1.005, 1.015) < 0.0001		
DBP (mmHg)				0.3167
< 90	10926	1.015 (1.012, 1.018)	< 0.0001	
≥ 90	965	1.012 (1.006, 1.018)	0.0002	

Above model adjusted for we adjusted age, sex, SBP, BMI, DBP, ALT, ALP, ALB, TBIL, GLB, UA, FBG, TG, Scr, and LDL-c. In each case, the model is not adjusted for the stratification variable. HR, Hazard ratios; CI, confidence; Ref, reference; NAFLD, non-alcoholic fatty liver disease.

### The results of the receiver operating characteristic curve analysis

**FIGURE 7:**
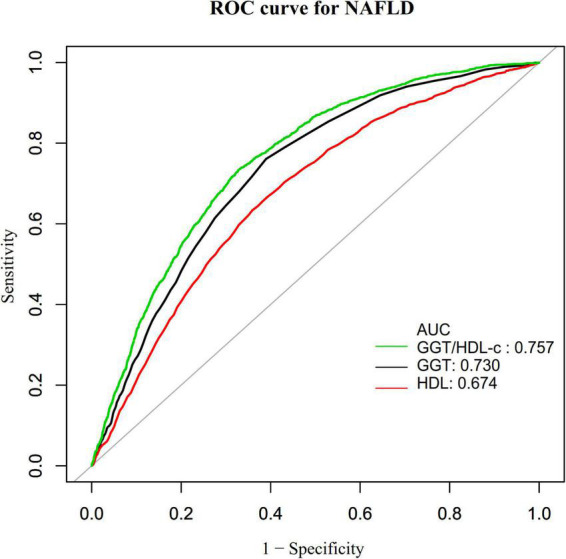
The results of receiver operating characteristic (ROC) curve analysis for measuring the ability of GGT, HDL-c, and GGT/HDL-c ratio to predict the risk of non-alcoholic fatty liver disease (NAFLD).

Additionally, we created a receiver operating characteristic (ROC) curve to assess the GGT, HDL-c, and GGT/HDL-c ratio capacity to predict the risk of NAFLD (Figure 6). The following table summarizes the areas under the curves for each variable: HDL-c: 0.674 < GGT: 0.730 < GGT/HDL-c ratio: 0.757 (Figure 7). The Youden index of GGT, HDL-c, and GGT/HDL-c ratio was 0.3712, 0.2745, 0.4055, and the corresponding optimal cut-off value was 22.5000, 1.335, 18.241, respectively. The Youden index and AUC of the GGT/HDL-c ratio was the largest, so the predictive ability of the GGT/HDL-c ratio to incident NAFLD was better than that of other variables (Supplementary Table 2).

## Discussion

The prospective cohort research was conducted to determine the association between the GGT/HDL-c ratio and NAFLD risk in non-obese individuals. We discovered that an increase in the GGT/HDL-c ratio was associated with a considerably higher risk of developing NAFLD. A threshold effect curve was also identified, and distinct associations between the GGT/HDL-c ratio and NAFLD on both sides of the inflection point. Furthermore, TG was identified as a possible impact modifier capable of altering the GGT/HDL-c ratio and NAFLD connection. Significantly stronger connections were identified in those with TG < 1.7 mmol/L, while weaker associations were detected in individuals with TG ≥ 1.7 mmol/L.

We found few studies currently investigating the relationship between the GGT/HDL-c ratio and the incidence of NAFLD through a literature search. A cross-sectional research conducted in China included 7,882 individuals who had a routine physical examination, with 14.5% diagnosed with NAFLD. After adjusting for pertinent variables, the results indicated that the GGT/HDL-c ratio was positively linked with NAFLD (OR = 1.003, 95% CI 1.001–1.006) (18). We found a stronger relationship between GGT/HDL-c ratio and NAFLD, which are inconsistent with previous studies. Further differences include the following: (i) The study population differed. Their study focused on people who had health check-ups over a while, while our study focused on non-obese people who had health check-ups in hospitals. (ii) The study design and methodology for analyzing the link between the GGT/HDL-c ratio and NAFLD were varied. Our study was prospective cohort study, whereas theirs was cross-sectional. (iii) They did not attempt to investigate the non-linear relationship between GGT/HDL-c ratio and NAFLD. (iv) Adjusted variables had also been different. Compared to the previous study, we had a larger sample size. Meanwhile, the sensitivity analysis found that this relationship still exists among participants without FPG > 6.1 mmol/L, ALT > 40 U/L, TG ≥ 1.7 mmol/L, or eGFR < 60 mL/min^⋅^1.73 m^2^. The efforts started before have proven the stability of the link between the GGT/HDL-c ratio and the probability of developing NALFD. The findings established a baseline for therapeutic intervention to decrease the risk of NAFLD by lowering the GGT/HDL-c ratio. In addition, we used a ROC curve to determine the capacity of GGT, HDL-c, and the GGT/HDL-c ratio to predict the risk of NAFLD, and discovered that the GGT/HDL-c ratio had the more predictive potential for NAFLD than either GGT or HDL-c alone. An elevated GGT/HDL-c ratio alerts people to a high risk of developing NAFLD during follow-up, alerting people to adjust their lifestyle habits in advance to reduce the incidence of NAFLD.

Why higher GGT/HDL-c ratios were associated with NAFLD remains unclear; however, two hypotheses could explain this phenomenon. First, studies have shown that elevated GGT is associated with insulin resistance and metabolic syndrome (47). Insulin resistance plays a vital role in NAFLD (48, 49). Therefore, GGT may affect the development of NAFLD by mediating insulin resistance. In addition, oxidative stress plays a fundamental role in the initiation and progression of NAFLD (50). Thus, HDL-c anti-oxidative activity might contribute to NAFLD pathogenesis (17). With high GGT/HDL-c ratios, high GGT with low HDL-c levels may be related to NAFLD.

A non-linear association between the GGT/HDL ratio and NAFLD risk was also discovered in this investigation. Using a two-piecewise Cox proportional hazards regression model, the current study found a non-linear relationship between the GGT/HDL-c ratio and NAFLD risk. After correcting confounding variables, the GGT/HDL-c ratio inflection point was 20.35. It showed that when GGT/HDL-c ratio was below 20.35, a 1 unit increase in the GGT/HDL-c ratio was associated with an 11.3% greater incidence rate of NAFLD. However, when GGT/HDL-c ratio > 20.35, a 1 unit increase in GGT/HDL-c ratio was associated with a 0.3% greater risk of NAFLD. We observed that, compared to participants with the GGT/HDL-c ratio > 20.35, participants with a GGT/HDL-c ratio ≤ 20.35 generally were younger and had lower BMI, ALT, TBIL, UA, FPG, TC, TG, BUN, Scr, LDL-c, and (Supplementary Table 1). However, the earlier factors were strongly associated with NAFLD (20, 51–55). When the GGT/HDL-c ratio was more remarkable than 20.35, the GGT/HDL-c ratio had a negligible influence on NAFLD. On the other hand, when the GGT/HDL-c ratio was less than 20.35, the risk factors for NAFLD were reduced, and their influence on NAFLD was lessened. At this point, the GGT/HDL-c ratio had a considerably enhanced effect. The curvilinear association between GGT/HDL-c and NAFLD has significant clinical implications. On the one hand, the risk of NAFLD was lower when we controlled the GGT/HDL-c ratio of non-obese people to below 20.35 through interventions. In addition, when the GGT/HDL-c ratio was below 20.35, the risk of NAFLD decrease more rapidly as the GGT/HDL-c ratio decrease. This provides a reference for optimizing NAFLD prevention decision-making and promoting clinical consultation.

The strengths of this study are as follows. (i) We had a relatively large sample size. (ii) To our knowledge, this is the first time that non-obese Chinese adults have been employed as a research cohort to examine the link between GGT/HDL-c and NAFLD. (iii) Compared with the previous research, the research on the non-linearity addressing is significantly improved. (iv) Multiple imputations were employed to handle missing data. This method can maximize statistical power and minimize potential bias caused by missing covariate information. Finally, we examined the robustness of the results in this study by performing a series of sensitivity analyses (conversion of the target-independent variable form, subgroup analysis, using a GAM to insert the continuity covariate as a curve into the equation, calculating *E*-values to investigate the possibility of unmeasured confounding, and reanalyzing the relationship between GGT/HDL-c ratio and NAFLD after excluding participants eGFR < 60 ml/min/1.73 m^2^, FPG > 6.1 mmol/L, TG ≥ 1.7 mmol/L, or ALT > 40 U/L) to ensure the reliability of the results.

Our study also has limitations. First, the study design is observational, so the causal relationship could not be exactly determined due to the nature of the observational study design. Second, the findings are generalizable to non-obese Chinese adults with a normal LDL-c range. Third, the connection between GGT/HDL-c and NAFLD may vary among subjects with BMI > 25 kg/m^2^ or LDL-c > 3.12 mmol/L. In the future, we can consider designing our studies and enrolling all individuals, collecting all the participants, including normal weight and obese patients, with normal and abnormal LDL-c levels. The fourth caveat is that unmeasured confounders may still exist, as with any observational study, even if known potential confounders were adjusted for. However, we estimated the *E*-value to assess the possible influence of unmeasured confounding variables and determined that unmeasured confounding variables were unlikely to explain the results. Besides, the current study assessed GGT, HDL-c, and other parameters at baseline and did not take into account changes in the GGT/HDL-c ratio over time. Finally, ultrasound is not the gold standard for the diagnosis of NALFD due to its imperfect sensitivity. However, this study was based on a physical examination survey of an asymptomatic population with a large sample size, which usually does not allow for invasive liver biopsies and expensive and complex tests, such as ultrasound transient elastography and proton magnetic resonance spectroscopy (MRS) magnetic resonance imaging (MRI). In the future, we can design our own studies to diagnose NAFLD with more appropriate methods, such as elastography.

Translated with www.DeepL.com/Translator (free version).

## Conclusion

This study reveals a positive and non-linear relationship between the GGT/HDL-c ratio and NAFLD in non-obese Chinese with a normal LDL-c. Furthermore, there is a threshold impact between the GGT/HDL-c ratio and NAFLD. When the GGT/HDL-c ratio is less than 20.35, the GGT/HDL-c ratio is substantially related to the NAFLD risk. Therefore, the present study recommends optimizing NAFLD prevention decision-making and promoting clinical consultation.

## Data availability statement

Publicly available datasets were analyzed in this study. This data can be found here: https://doi.org/10.5061/dryad.1n6c4.

## Ethics statement

The studies involving human participants were reviewed and approved by the Ethics Committee of Wenzhou People’s Hospital. Written informed consent for participation was not required for this study in accordance with the national legislation and the institutional requirements.

## Author contributions

QL, YH, and HH conceived the research drafted the manuscript and Performed the statistical analysis. YZ revised the manuscript and designed the study. All authors have read and approved the final manuscript.

## Footnotes

1
www.datadryad.org

## Abbreviations

ALB: Albumin
ALP: Alkaline phosphatase
ALT: Alanine aminotransferase
AST: Aspartate aminotransferase
BMI: Body mass index
BUN: Serum urea nitrogen
CI: Confidence intervals
CKD: Chronic kidney disease
DBIL: Direct bilirubin
DBP: Diastolic blood pressure
eGFR: Estimated glomerular filtration rate
FPG: Fasting plasma glucose
GAM: Generalized additive model
GGT: Gamma-glutamyl transferase
GLB: Globulin
HDL-c: High-density lipoprotein cholesterol
HR: Hazard ratio
HTG: Hypertriglyceridemia
IFG: Impaired fasting glucose
LDL-c: Low-density lipoprotein cholesterol
METS: Metabolic syndrome
NASH: Non-alcoholic steatohepatitis
NAFLD: Non-alcoholic fatty liver disease
Ref: Reference
SBP: Systolic blood pressure
Scr: Serum creatinine
SD: Standard deviation
T2DM: Type 2 diabetes mellitus
TBIL: Total bilirubin
TC: Total cholesterol
TG: Triglyceride
UA: Uric acid
WHO: World Health Organization.
