# Supplementary material for: Gamma-glutamyl transferase to high-density lipoprotein cholesterol ratio has a non-linear association with non-alcoholic fatty liver disease: A secondary prospective cohort study in non-obese Chinese adults
Source: Front Med (Lausanne). 2022 Nov 17;9:995749. doi: 10.3389/fmed.2022.995749 (PMC9712445; doi:10.3389/fmed.2022.995749)
Supplement: Supplementary file 1 [file Data_Sheet_1.pdf]

Gamma-glutamyltransferase to high-density lipoprotein cholesterol ratio has a non-linear association with non-alcoholic fatty liver disease: a secondary prospective cohort study in the non-obese Chinese population.

Running title: GGT/HDL-c ratio and NAFLD

Qiming Li[1],Yong Han[2], Haofei Hu[3], Yuzheng Zhuge [1]*

1 Department of Gastroenterology, Nanjing Medical University Drum Tower Clinical Medical School, Nanjing, Jiangsu, China

2 Department of Emergency, Shenzhen Second People's Hospital, Shenzhen 518035, Guangdong Province, China

3 Department of nephrology, Shenzhen Second People's Hospital, Shenzhen 518035, Guangdong Province, China

*Corresponding author

Yuzheng Zhuge[1]

Department of Gastroenterology, Nanjing Medical University Drum Tower Clinical Medical School

No.321, Zhongshan Road, Gulou District,

Nanjing 210008,

Jiangsu Province,

China

Tel: +86-025-83105206, +86-15996289206

Fax: +86-025-83304616

Email: yuzheng9111963@aliyun.com

TableS1. The characteristics of participants on both sides of the inflection point.

GGT/HDL-c ratio	<20.35	>=20.35	P-value
N	7937	3954	
SEX			<0.001
Female	3741 (47.13%)	1648 (41.68%)	
Male	4196 (52.87%)	2306 (58.32%)	
Age(years)	43.02 ± 15.03	43.84 ± 14.78	0.005
ALP(U/L)	68.29 ± 20.03	78.94 ± 24.54	<0.001
ALT(U/L)	16.36 ± 9.58	25.96 ± 21.55	<0.001
AST(U/L)	21.23 ± 6.34	25.75 ± 11.84	<0.001
ALB(g/L)	44.48 ± 2.76	44.67 ± 2.80	<0.001
GLB(g/L)	29.19 ± 3.92	29.47 ± 4.11	<0.001
TBIL(umol/L)	12.08 ± 4.90	12.63 ± 5.34	<0.001
DBIL(umol/L)	2.20 ± 1.16	2.22 ± 1.27	0.904
BUN (umol/L)	4.50 ± 1.36	4.75 ± 1.50	<0.001
Scr (umol/L)	80.10 ± 19.64	90.12 ± 33.74	<0.001
UA (umol/L)	269.38 ± 83.76	336.43 ± 80.71	<0.001
FPG(mmol/L)	5.12 ± 0.67	5.38 ± 1.05	<0.001
TC(mmol/L)	4.57 ± 0.72	4.66 ± 0.75	<0.001
TG(mmol/L)	1.10 ± 0.49	1.84 ± 1.12	<0.001
LDL-c(mmol/L)	2.24 ± 0.47	2.34 ± 0.46	<0.001
BMI(kg/m^2)	21.15 ± 2.02	22.47 ± 1.77	<0.001
SBP(mmHg)	119.67 ± 16.63	127.33 ± 16.32	<0.001
DBP(mmHg)	72.07 ± 9.98	76.87 ± 10.30	<0.001

Values are n (%) or mean ± SD or median (quartile)

BMI, Body mass index; DBP, Diastolic blood pressure; ALP, Alkaline phosphatase; SBP, Systolic blood pressure; AST, Aspartate aminotransferase; TG, Triglyceride; ALB, albumin; ALT, Alanine aminotransferase; GLB, globulin; LDL-c, Low-density lipid cholesterol; BUN, Serum urea nitrogen; Scr, Serum creatinine; TC, Total cholesterol; FPG, Fasting plasma glucose; UA, uric acid; DBIL, Direct bilirubin; TBIL, Total bilirubin. GGT/HDL-c ratio, Gamma -glutamyl transpeptidase to high-density lipoprotein cholesterol ratio.

TableS2. AUC of GGT, HDL-c, TG, and GGT/HDL-c ratio for predicting NAFLD

Variable	AUC	Best threshold	Specificity	Sensitivity	Yorden Index
GGT	0.730	22.5000	0.6104	0.7608	0.3712
HDL-c	0.674	1.3350	0.6409	0.6336	0.2745
GGT/HDL-c ratio	0.757	18.2411	0.6723	0.7332	0.4055

AUC: area under the curve; GGT, gamma-glutamyl transferase; HDL-c high-density lipoprotein cholesterol ;GGT/HDL-c ratio, Gamma - glutamyl transpeptidase to high-density lipoprotein cholesterol ratio.

Figure S1. Incidence of NAFLD according to the quartiles of eGFR.

FigureS1. Participants in the high GGT/HDL-c ratio group had a higher NAFLD incidence than the lowest GGT/HDL-c ratio group ($p<0.001$ for trend).
